# Radiographic Peri‐Implant Bone Changes in Osteoporotic Women Treated With a Ti‐Zr, Bone Level Tapered Implant With a Hydrophilic Surface: A 12‐Month Prospective Case‐Series

**DOI:** 10.1111/clr.14469

**Published:** 2025-07-08

**Authors:** Calciolari Elena, Mardas Nikos, Palaska Iro, Tagliaferri Sara, Donos Nikolaos

**Affiliations:** ^1^ Centre for Oral Clinical Research, Institute of Dentistry, Faculty of Medicine and Dentistry Queen Mary University of London London UK; ^2^ Department of Medicine and Surgery University of Parma Parma Italy

**Keywords:** bone regeneration, dental implantation, osseointegration, osteoporosis, post‐menopausal

## Abstract

**Objectives:**

This study assessed 12‐month post‐loading 3D peri‐implant radiographic bone changes in osteoporotic women receiving a single titanium‐zirconium bone‐level tapered dental implant with a hydrophilic surface.

**Materials and Methods:**

This was a prospective case series involving 18 post‐menopausal osteoporotic women in need of a single dental implant. A standardized CBCT scan was performed after implant placement and at 12 months post‐loading to assess peri‐implant bone changes. The Implant Stability Quotient (ISQ) was recorded after implant placement, at implant impression, loading, and 12 months post‐loading. Peri‐implant clinical parameters were recorded at 6 and 12 months post‐loading and during the last visit implant survival and success were also evaluated.

**Results:**

Seventeen patients completed all study visits, and implant placement was uneventful for all participants. A statistically significant difference (reduction) from implant placement to 12 months post‐loading was observed in terms of radiographic buccal bone width and palatal/lingual width (ΔBw‐0: 0.53 mm, *p* < 0.001 and ΔPw‐0: 0.47 mm, *p* = 0.006), as well as in terms of vertical distance between the implant shoulder and the first bone to implant contact on the buccal and palatal/lingual aspect (ΔBICb: −0.26 mm, *p* = 0.005 and ΔBICp: −0.46 mm, *p* = 0.018). ISQ increased during osseointegration, and a high implant survival (100%) and success rate (from 81.3% to 100% based on 3 different sets of criteria) were recorded at 12 months post‐loading.

**Conclusion:**

In this case series, osteoporotic patients treated with single titanium‐zirconium implants showed high survival rates, predictable 12‐month implant outcomes, and physiologic peri‐implant bone remodelling.

**Trial Registration:** NCT02884401

## 1 Introduction

Osteoporosis is a common skeletal disease characterized by a progressive reduction in bone mass and changes in the micro‐architectural structure of bone, which dramatically increase the risk of fractures (Consensus development conference: Diagnosis, prophylaxis, and treatment of osteoporosis 1993). Women after menopause are the most affected, since oestrogen deficiency has been associated with increased formation, recruitment, and activity of osteoclasts through several local cytokines and growth factors (Gallagher 2001; McLean 2009), decreased survival of osteocytes, impaired response of osteoblasts to mechanical stimuli, and bone micro‐damage (Ejiri et al. 2008; Nicks et al. 2010). Owing to population growth and ageing, it is expected that the number of osteoporotic patients will significantly increase (Salari et al. 2021), hence the number of osteoporotic patients requiring dental care, including implant‐related rehabilitations, is expected to rise.

It is biologically plausible that the alterations in bone metabolism associated with osteoporosis can also impair bone healing around dental implants and negatively affect their osseointegration. As a matter of fact, previous systematic reviews indicated a moderate correlation between jawbone and skeletal bone mineral density (BMD) (Calciolari et al. 2016) and that osteoporosis does manifest in the jawbones so that it can even be detected through specific indices assessed on dental panoramic radiographs (Calciolari et al. 2015).

Pre‐clinical studies overall suggest a lower osseointegration rate and reduced mechanical properties in osteoporotic bone (Dereka et al. 2018; Donos et al. 2015), while clinical evidence is less robust. The efficacy of dental implants in osteoporotic patients has been assessed in prospective case–control studies, and overall, they support the applicability of implants in osteoporotic patients, even for immediate loading (Degidi and Piattelli 2003). Therefore, nowadays a diagnosis of osteopenia or osteoporosis is not considered an absolute contraindication to dental implants (Amorim et al. 2007; Dao et al. 1993; Friberg et al. 2001; Holahan et al. 2008, 2011; Tsolaki et al. 2009). Nevertheless, there are data mainly coming from retrospective analyses indicating that osteoporosis might be associated with a lower implant success rate, especially in the case of an implant placed in augmented bone (Kramer et al. 1999; Pinholt 2003; Schliephake et al. 1997).

One possibility to enhance speed and quality of osseointegration is to modify implant surface properties (such a topography, wettability, charge, and chemistry). Sandblasting, Large‐grit, Acid‐etching (SLA) is a commonly applied technique to increase surface roughness, which has been shown to upregulate genes associated with wound healing and regeneration, mesenchymal stem cell differentiation, and skeletal development, including upregulating the Wnt pathway (Donos, Retzepi, et al. 2011) as compared to machined surfaces. Chemical modification of the SLA implant surfaces to increase surface free energy and wettability (SLActive) and its subsequent hydrophilicity has also been tested to further strengthen the osteogenic properties of this surface. The rationale behind this is that a hydrophilic surface should promote interactions with cells and tissues at the initial healing stages, when there is protein adsorption on the surface and cell adhesion, thus further promoting osteogenesis (Rupp et al. 2006; Wall et al. 2009; Yahyapour et al. 2004), particularly during the early healing period (Bornstein et al. 2008; Buser et al. 2004; Ferguson et al. 2006). We previously described the genes (Donos, Hamlet, et al. 2011) and proteins (Calciolari, Mardas, et al. 2018) expressed during bone formation and osseointegration associated with hydrophobic and hydrophilic microrough surfaces, and we showed that hydrophilic surfaces upregulate pathways coupling osteogenesis and angiogenesis, and downregulate inflammation‐associated pathways (Calciolari, Hamlet, et al. 2018).

Remarkably, a previous pre‐clinical study showed that a hydrophilic microrough surface might be able to reverse the negative impact of osteoporosis on bone formation and osseointegration (Mardas et al. 2011), thus making dental implants in osteoporotic patients a more predictable treatment option. A possible biological explanation for this outcome lies in the fact that osseous healing in osteoporotic conditions has been associated with a tendency for an enhanced inflammatory and stress response and a delayed organization and maturation of the granulation tissue (Calciolari, Mardas, Dereka, Anagnostopoulos, et al. 2017; Calciolari, Mardas, Dereka, Kostomitsopoulos, et al. 2017). Since a hydrophilic surface is able to modulate inflammatory and osteogenic‐related pathways, it might be particularly beneficial in osteoporotic patients, where the same pathways are negatively affected.

Clinical studies have confirmed positive short‐term outcomes when such implants were placed in challenging situations, such as in irradiated patients (Heberer et al. 2011; Nack et al. 2015), in patients with poorly controlled type II diabetes (Khandelwal et al. 2013) and in immediate and early implant loading in the posterior maxilla/mandible (Bornstein et al. 2009; Ganeles et al. 2008; Morton et al. 2010; Roccuzzo and Wilson Jr. 2009; Salvi et al. 2004). To the best of our knowledge, the possibility of using micro‐rough hydrophilic implants in osteoporotic bone has not been investigated in human studies yet. Therefore, this study aimed to document the use of dental implants with a hydrophilic surface for single tooth replacement in a cohort of osteoporotic patients and to assess the radiographic stability of peri‐implant bone levels at up to 12 months post‐loading.

## 2 Materials and Methods

This is a prospective case series that aimed to radiographically evaluate the vertical and horizontal alveolar bone changes 12 months after implant loading (primary outcome) in 18 post‐menopausal women in need of a single tooth replacement with a dental implant. The secondary objectives were to assess implant stability by applying resonance frequency analysis and to evaluate implant success and survival 12 months after loading.

The study was approved by the London Bridge Research Ethics Committee on 29th April 2016 (16/LO/0477) and registered in clinicaltrials.gov (NCT02884401). It consisted of 8 visits performed within a minimum period of 15 months at the Centre for Oral Clinical Research (COCR) at Barts Health NHS Trust Dental Hospital, Queen Mary University of London. Participants were recruited from the Rheumatology Department, DXA Clinic, and Dental Hospital at Barts Health NHS Trust. Leaflets advertising the study and giving contact points were placed in the Rheumatology and Radiology Departments, as well as in the Dental Hospital at Barts Health NHS Trust. The study was also advertised with the Royal Osteoporosis Society via its media streams.

This case series has been reported in line with the PROCESS guideline.

### 2.1 Inclusion and Exclusion Criteria

Each participant met the following inclusion criteria to be enrolled in the study:

- Diagnosis of osteoporosis based on dual‐energy x‐ray absorptiometry (DXA) measurement of BMD at the femur neck and/or total hip and/or lumbar spine (T value 2.5 SD or more below the young female adult mean) within the past 24 months.
- Not in treatment with anti‐resorptive agents (like bisphosphonates and denosumab) for more than 4 years, to reduce the risk of medication‐related osteonecrosis of the jaws (Lo, et al. 2010).
- ≥ 50 years old.
- In self‐reported menopause, defined as the permanent cessation of ovulation, for at least one year (Soules et al. 2001).
- Edentulous area involving a maximum of two teeth (wisdom teeth and second molars are excluded) and presenting at least one neighboring tooth (e.g., gap in the area of a second premolar and first molar, with first premolar in place).
- Residual alveolar width ≥ 4 mm at bone level (Milinkovic and Cordaro 2014), residual alveolar height > 8 mm, enough inter‐arch space for a crown (at least 5 mm), and a minimum distance of 7 mm from the adjacent teeth (Shah and Lum 2008). The width and height were confirmed after x‐ray examination in Visit 2.
- Possibility to restore a functional occlusion with a minimum of four occlusal units (i.e., pairs of occluding posterior teeth).
- Willingness to replace the missing tooth/teeth with dental implants
- Registration with a general dental practitioner.

Participants were not eligible for participation in the study if:

- On chronic treatment (i.e., two weeks or more) with any medication severely affecting oral status (e.g., participants with gingival hypertrophy caused by anti‐epileptics, calcium antagonists, cyclosporine and other immunosuppressive) or bone metabolism (e.g., anticoagulant medications, long‐standing steroid medications—i.e. equal or more 2.5 mg of prednisolone a day taken for more than 3 months ‐, anticonvulsants, immunosuppressants).
- Affected by systemic diseases recognized to severely affect bone metabolism (e.g., Cushing's syndrome, Addison's disease, diabetes mellitus type 1, leukaemia, pernicious anaemia, malabsorption syndromes, chronic liver disease, rheumatoid arthritis).
- Knowingly affected by HIV or viral hepatitis.
- Having a history of local radiation therapy in the last five years.
- Affected by limited mental capacity or language skills such that study information cannot be understood, informed consent cannot be obtained, or simple instructions cannot be followed.
- Presenting an acute endodontic/periodontal lesion in the neighbouring areas to the implant site.
- Presenting severe bruxism or clenching habits (self‐reported)
- Smoking > 5 cigarettes a day.
- Having a daily alcohol intake > 2 units/day.

### 2.2 Experimental Protocol

**FIGURE 1:**
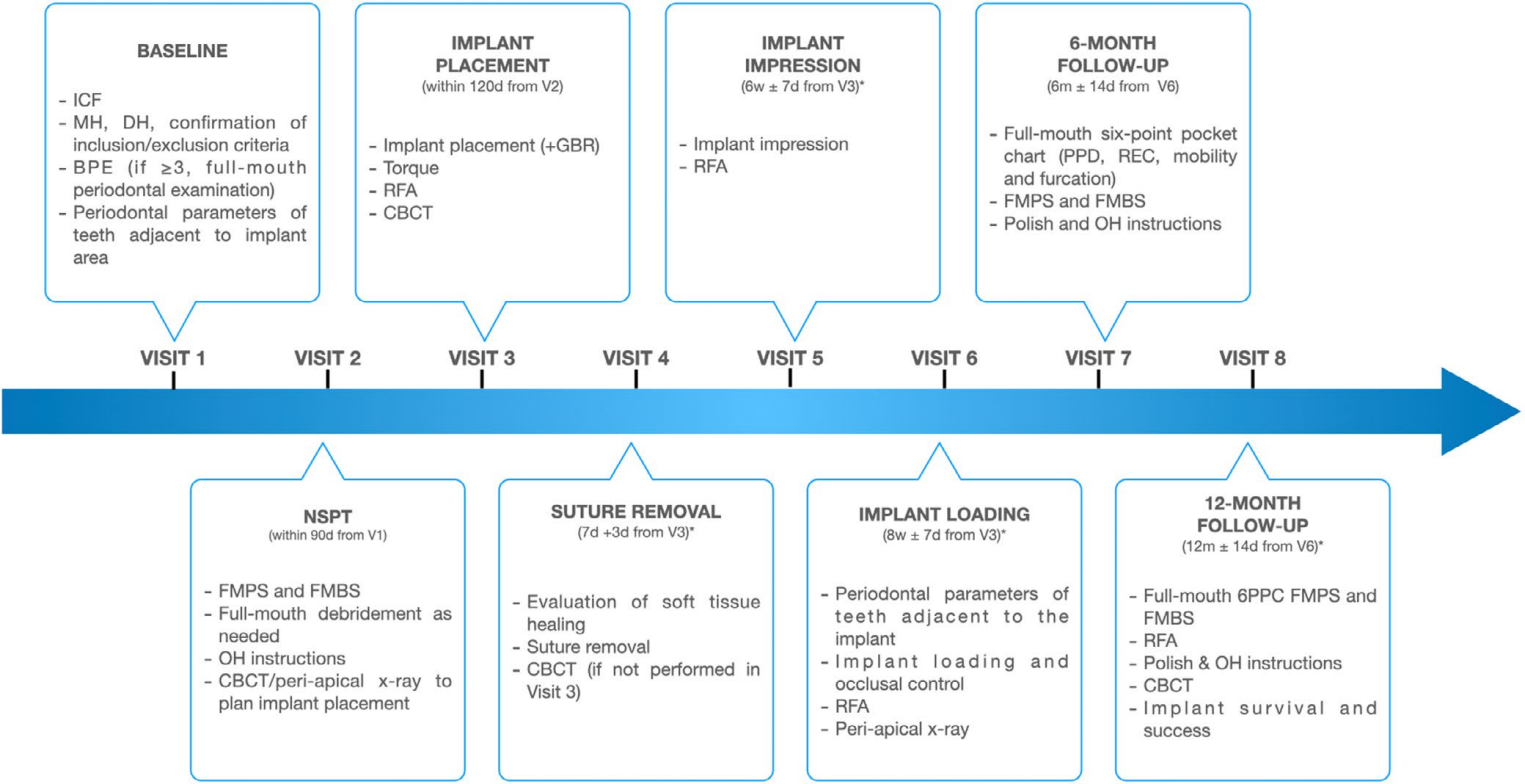
Flowchart of study visits. 6PPC, six‐point pocket chart; DH, dental history; FMBS, full‐mouth bleeding score; FMPS, full‐mouth plaque score; ICF, informed consent form; MH, medical history; OH, oral hygiene; RFA, resonance frequency analysis. *Whenever GBR was performed, suture removal was done at 14 days ± 5 days after implant placement, and implant impression and loading were performed at 11 weeks (+7 days) and 12 weeks (+7 days) after implant placement, respectively.

Details about study visits are summarized in Figure 1. Briefly, during the enrollment/baseline visit (Visit 1), a detailed medical and dental history was recorded, as well as the periodontal parameters adjacent to the tooth to be replaced, including probing pocket depth (PPD), recession (REC), plaque, and bleeding score. In case of a basic periodontal examination (BPE) ≥ 3 in any sextant, a full‐mouth six‐point pocket chart was recorded, which included PPD, REC, furcation involvement, mobility, and recording of full‐mouth plaque and bleeding scores (FMPS and FMBS). Within 90 days from enrollment, a second visit was performed to check FMPS and FMBS and provide non‐surgical debridement and oral hygiene instructions, as needed. In case a participant required further periodontal treatment (non‐surgical or surgical), this was arranged outside the study protocol (after Visit 1 and/or Visit 2) until a condition of periodontal stability (no pockets > 5 mm, FMPS and FMBS < 25%) was reached.

A peri‐apical x‐ray or cone‐beam computed tomography (CBCT) scan was performed to adequately plan implant placement, which was then performed by two experienced periodontists with more than 20 years of clinical activity within 120 days from visit 2. After performing local anesthesia, a crestal incision and a full thickness flap elevation with preservation of as much soft tissue as possible was performed extending mesially and distally to the adjacent teeth. A tapered, bone‐level, titanium‐zirconium alloy implant (Straumann BLT Roxolid SLActive, Basel, Switzerland) was then inserted according to the manufacturer's guidelines, aiming to achieve primary stability in the correct prosthetic position. The diameter and the length of the implant were selected according to the clinical indication of the site and based on previous radiographic evaluation. The torque achieved at insertion was recorded, and implant stability was assessed through resonance frequency analysis (RFA).

In case of buccal fenestration/dehiscence, GBR was simultaneously performed by loosely compacting a bovine osteoconductive graft (Cerabone, Botiss, Zossen, Germany) on the buccal defect, which was then covered with a non‐cross‐linked collagen membrane (Collprotect, Botiss, Zossen, Germany). GBR was performed in a way to slightly overbulk the alveolar bone profile.

A semi‐submerged healing of the relevant healing cap (conical shape Ti healing abutment, Straumann, Basel, Switzerland) was achieved, and the flap was coronally positioned to fully cover the regeneration materials. In case the bone quality was considered poor or torque was < 15 N/cm, a submerged healing was obtained. No sinus floor augmentation cases were considered. A standardized CBCT scan was performed either at the end of implant surgery or at suture removal, which took place one week later (or 2 weeks in case GBR took place).

Post‐operative pain and oedema were controlled with paracetamol (2 × 500 mg for the first couple of days or more if needed) or other painkillers, such as ibuprofen (2 × 400 mg for the first couple of days or more if needed) according to the medical history of the patient. All patients received systemic antibiotics as follows: amoxicillin 1 g 1 h before the surgery and then 500 mg every 8 h for 1 week. In case of allergy to amoxicillin, clindamycin (600 g 1 h before the surgery and then 300 mg every 6 h for 1 week) or erythromycin (1 g 1 h before the surgery and then 500 mg every 12 h for 1 week) was prescribed. The incidence of post‐operative adverse events and the quantity of painkiller intake were recorded.

Patients were instructed to rinse twice daily with 0.2% chlorhexidine digluconate during the first 4 weeks and to use modified oral hygiene procedures in the treated area for the first 6 post‐operative weeks. A soft diet was recommended for the first post‐operative week and to avoid placing food in the area of the surgery during the first 6 weeks.

An implant impression was performed 6 weeks after surgery (11 weeks in case of GBR) with a silicon material, and during this visit, implant stability was assessed through RFA. The ceramic prosthetic reconstruction was loaded 8 weeks post‐operatively (± 7 days). In case that bone quality was considered inadequate or torque was < 15 N/cm, or GBR was performed, a longer healing period was considered (12 weeks). At loading, implant stability was assessed again through RFA, and a peri‐apical x‐ray was performed to confirm that the crown was seating correctly.

Unless the tooth to be replaced was an anterior tooth (from canine to canine) or there were specific aesthetic requirements, no provisional restoration was provided. Otherwise, either a removable denture or an adhesive Maryland bridge was manufactured, paying attention to remove it from the occlusion.

Follow‐up visits were scheduled at 6 and 12 months post‐loading to record clinical periodontal data, provide a polish, and oral hygiene instructions. Moreover, at 12 months, RFA was repeated, and a new standardized CBCT scan was performed. Implant survival and success were also recorded.

### 2.3 Peri‐Implant Radiographic Bone Levels

CBCT imaging was acquired with a Carestream 9300 CBCT scanner (Carestream Health, Rochester, NY, USA) in the area of interest (implant site including the 2 neighbouring teeth) during Visit 3 (or Visit 4) and Visit 8 with the following technical parameters: 90 kV acceleration voltage, 5 mA beam current, field of view (FOV) diameter of 5 × 5 cm, FOV height of 4 cm, 360° rotation, and voxel size of 0.125 mm^3^. The CBCT followed SEDENTEXCT guidelines on radiation protection and on the use of CBCT in dental and maxillofacial radiology (Commission 2012). The scan was processed with its embedded segmentation function to remove scattering defects and obtain the maximal quality possible.

In order to evaluate the stability of the peri‐implant bone from implant placement to 12 months after loading, the following parameters were assessed:

1. Palatal/lingual alveolar ridge width (Pw‐0): the thickness of the palatal/lingual bone measured as the distance from the alveolar bone crest (pristine or augmented) to the first bone to implant contact on a line perpendicular to the implant axis. Moreover, the palatal/lingual width at 1 (Pw‐1), 2 (Pw‐2), and 4 (Pw‐4) mm from the alveolar bone crest was also calculated.
2. Buccal alveolar ridge width (Bw‐0): the thickness of the buccal bone measured as the distance from the alveolar bone crest (pristine or augmented) and the first bone to implant contact on a line perpendicular to the implant axis. Moreover, the buccal width at 1 (Bw‐1), 2 (Bw‐2), and 4 (Bw‐4) mm from the alveolar bone crest was also calculated.
3. The vertical distance between the implant shoulder and the first bone to implant contact on the buccal (BICb) and palatal/lingual (BICp) side.

After randomly numbering the scans, a single, previously calibrated examiner (EC) took all the measurements 3 times at a distance of 48 h, and the average value was recorded. All measurements were calculated with a 0.1 mm precision level. Specific reference points were picked from the images in order to obtain the same 3D position of the measurement axis at implant placement (or at suture removal) and at 12 months post‐loading (Degidi et al. 2013) (Figure S1). Blinding was not feasible, since the CBCT performed at 12‐month follow‐up showed the implant crown in place, so it was always recognizable.

### 2.4 Implant Stability

Resonance frequency analysis (RFA) was applied to assess implant stability. In particular, the implant stability quotient (ISQ) was recorded after implant placement (Visit 3), at implant impression (Visit 5), loading (Visit 6), and at 12‐month post‐loading (Visit 8) with Osstell ISQ (Osstell, Gothenburg, Sweden). During each visit, ISQ was measured 3 times in mesio‐distal and bucco‐lingual directions, and the average values were recorded.

### 2.5 Implant Success and Survival

At 12 months post‐loading, implant survival and success were recorded. More specifically, in order to allow a more comprehensive assessment/description of implant success, 3 different sets of criteria were used. (Albrektsson et al. 1986; Buser et al. 1990; Ong et al. 2008).

### 2.6 Statistical Analysis and Sample Size

Since this is a prospective case series study and only a limited number of studies have addressed peri‐implant alveolar bone changes in post‐menopausal osteoporotic women, and no previous study has applied the same methodologies/techniques, a precise sample size calculation was not possible. The study team agreed that having 20 patients completing the study would allow us to retrieve enough data to power future larger studies, if needed.

Considering a potential drop‐out rate of 10%, we initially planned to recruit 22 post‐menopausal women for this trial. With a higher actual drop‐out rate experienced (6 patients dropped out of 22 patients enrolled), we subsequently amended the protocol to enable recruiting up to 30 patients.

Descriptive statistics were applied to summarize patients' demographics. Due to the lack of normality distribution and limited sample size, differences in peri‐implant vertical and horizontal bone level at implant placement and 12 months after loading were assessed with a non‐parametric test (Wilcoxon signed rank test). For the parameters that showed a significant change from baseline to 12‐months post loading (Δ, delta difference calculated as baseline—12‐months post loading values), correlation between the delta change with demographics, clinical parameters, performance of GBR, medical history, and T score was assessed through Spearman correlation coefficient. Due to the small sample size, a regression model was not developed.

For RFA, changes between time points in ISQ were assessed through the Friedman test, followed by the Dunn test, as a post hoc analysis (with Bonferroni's adjustment).

The 95% confidence intervals (CIs) for survival and success rates were calculated using the exact binomial (Clopper–Pearson) method (Python 3.11, Statsmodels v0.14, method = ‘beta’), which is appropriate for small sample sizes or when proportions are close to 0% or 100%.

Statistical analyses were performed through SPSS Statistics 25 (IBM Inc., Chicago, IL, USA). Statistical significance was set at *p* ≤ 0.05.

In case of withdrawn patients, if the last visit attended was before implant placement (visit 3), data were discarded since all primary and secondary outcomes required measurements performed after implant placement. If patients withdrew after visit 3, available data were analyzed up to the last visit attended.

## 3 Results

From September 2016 until July 2021, 27 post‐menopausal osteoporotic women were enrolled. Nine patients withdrew before Visit 3 either because they could not reach an adequate level of periodontal health (*n* = 1) or because the CBCT scan showed inadequate residual alveolar bone to plan implant placement without the need for sinus lift or staged GBR (*n* = 7). One patient dropped out for personal reasons that did not allow her to travel and attend study visits (*n* = 1). One patient withdrew after Visit 7 for personal reasons, so for this patient, data up to Visit 7 were analysed (intention‐to‐treat analysis). We decided to stop recruitment with 17 patients completing the study (instead of 20), since, due to the COVID‐19 pandemic, we encountered challenges in recruiting new patients and in having patients willing to travel to attend study visits. Considering that this is a case series, and the sample size was arbitrarily established, the team agreed that for the purposes of this project, the reduced number of patients would not have significantly impacted the outcomes.

Table 1 summarizes the demographics of the recruited patients. Briefly, patients had a mean age of 62.33 ± 6.08 years (min 53, max 75 years) and a mean T score of −2.91 ± 0.35 (min −3.7, max −2.5), with half of them having had already one or more fragility fractures (severe osteoporosis or stage IV). None of the patient was a current smoker, but 5 of them used to smoke in the past. The majority were of White ethnicity, with 2 patients from Asian ethnicity and 1 from Black/African American ethnicity. Ten patients were currently under treatment with osteoporosis medications (bisphosphonates) for less than 4 years, two patients had taken bisphosphonates in the past, and 6 patients had never been treated pharmacologically with osteoporosis medications. Six patients reported that they underwent periodontal treatment in the past.

**TABLE 1:** Demographics and general characteristics of the patients.

Age (mean ± SD; min–max)	62.33 ± 6.08; 53–75
T score (mean ± SD; min‐max)	−2.91 ± 0.35; −3.7 to 2.5
Ethnicity (%, number)	White: 83.3% (15) Asian: 11.1% (2) Black or African American: 5.6% (1)
Smoking status (%, number)	Smoker: 0% Past smoker: 27.8% (5) Non‐smoker: 72.8% (13)
Osteoporosis medications (%, number)	Past use: 11.11% (2) Current use: 55.56% (10) No medication: 33.33% (6)
Implant site (%, number)	Incisors: 11.1% (2) Canines: 5.6% (1) Premolars: 33.3% (6) Molars: 50% (9)
	Maxilla: 50% (9) Mandible: 50% (9)
Previous periodontal treatment (%, number)	33.3% (6)

In all 18 patients that received a dental implant, surgeries were uneventful, but in two patients, implants showed spinning when screwing the healing abutment; hence, they were submerged. Five patients required GBR concomitant to implant placement, and in all of these cases, it was decided to submerge the implant. The dehiscences/fenestrations had a width ranging from 0 to 4 mm and a height ranging from 0 to 12 mm. Insertion torque was ≤ 15 Ncm in four implants. No post‐surgical adverse events outside the ones normally expected for a minor oral surgery were recorded after implant placement. In particular, 7 patients reported minor pain/discomfort and swelling on the days following implant surgery, and 3 also experienced headaches. Three patients developed a small bruise on the cheek/lip, and one of these also experienced minor vertigo. While 7 patients only took painkillers on the day of the surgery, 11 patients took additional painkillers (namely paracetamol) for a period ranging from 1 to 7 days after the surgery. Local signs of inflammation were recorded at suture removal in 4 patients, but they were easily controlled with the use of local antiseptics. One patient experienced a serious adverse event not related to the study (broken ulna that required surgery and hospitalization).

**FIGURE 2:**
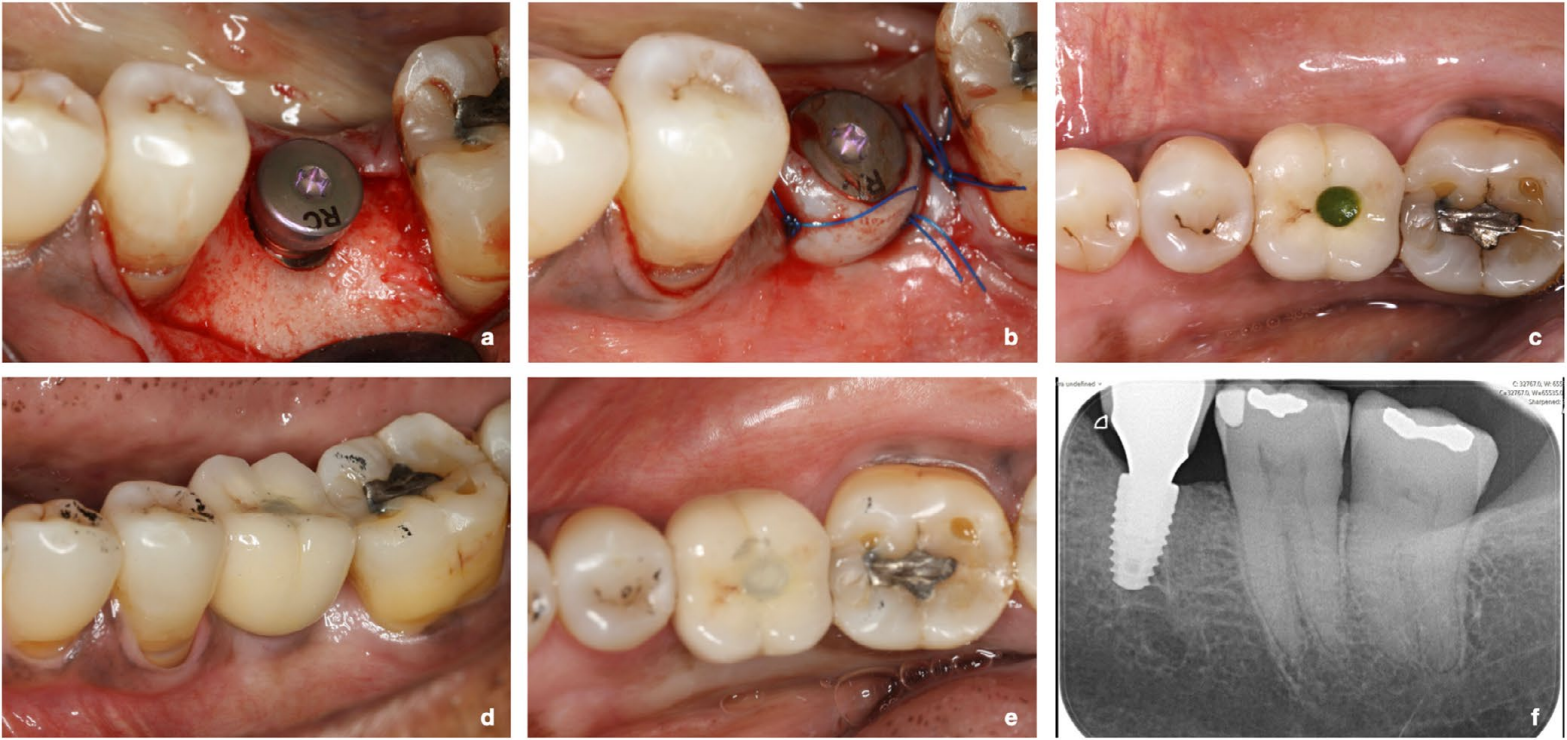
Sequence of treatment for the replacement of a lower left first molar. (a) Implant placed and healing abutment screwed; (b) Suture of the flap around the healing abutment (transmucosal healing); (c, d) Implant loading; (e, f) Occlusal check and peri‐apical x‐ray at 1 year post loading.

Figure 2 shows an example of a case successfully treated as part of this study.

### 3.1 Radiographic Peri‐Implant Bone Levels

**FIGURE 3:**
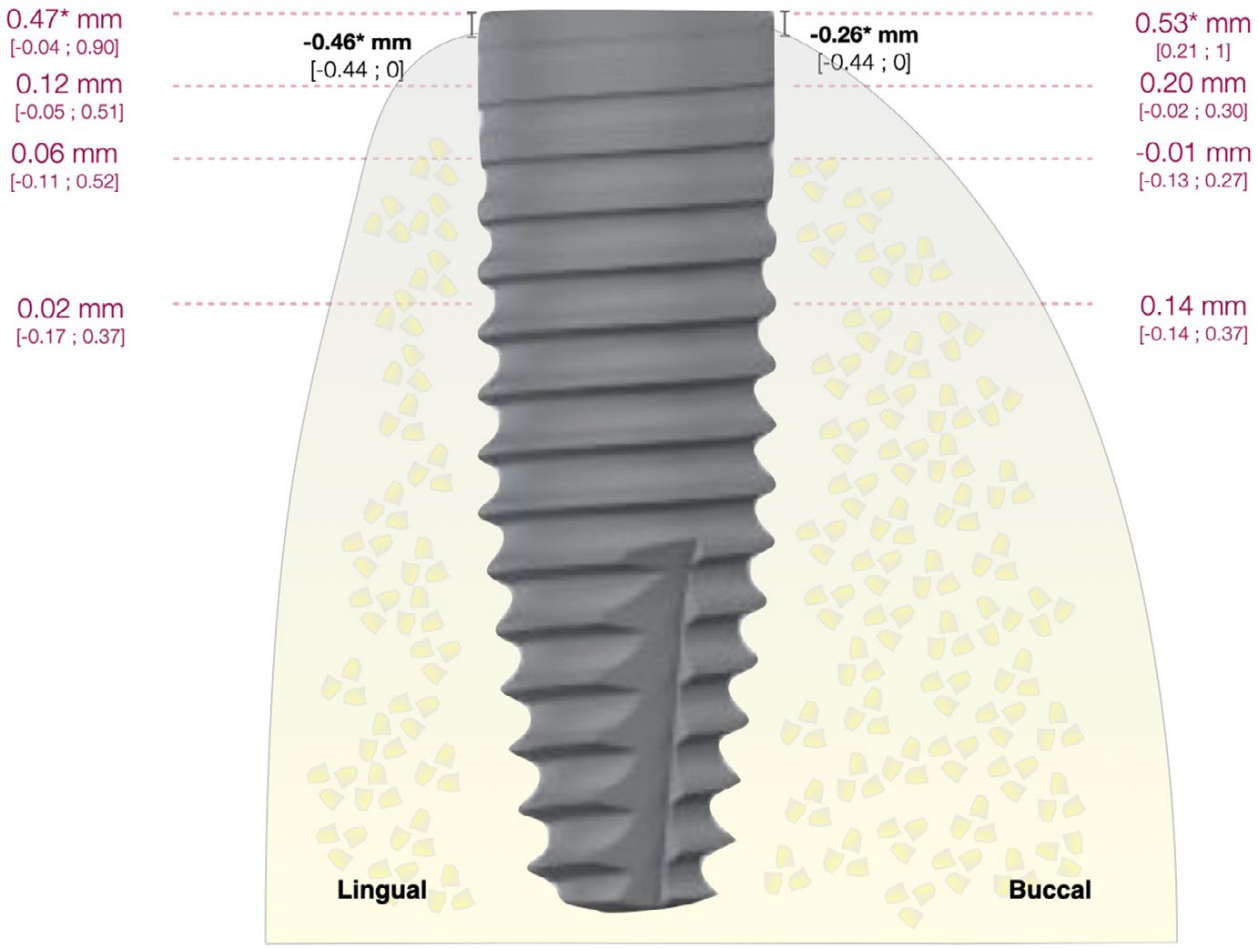
Graphical representation of the mean changes (Δ) in buccal and lingual peri‐implant bone levels from implant placement to 12 months post‐loading, as assessed through CBCT scans. Buccal width changes were assessed at the level of the implant platform, at 1, 2, and 4 mm from the implant platform. *Identifies statistically significant differences from implant placement to 12 months post‐loading. Data are presented as median [interquartile range].

The median and inter‐quartile range of radiographic peri‐implant bone levels at implant placement and at 12 months post‐loading are reported in Table 2. A statistically significant difference from implant placement to 12‐month post‐loading was observed in terms of buccal width and palatal/lingual width at the level of implant platform (ΔBw‐0: 0.53 mm; 0.21–1, *p* < 0.001 and ΔPw‐0: 0.47 mm; −0.04 to 0.90, *p* = 0.006), as well as in terms of vertical distance between the implant shoulder and the first bone to implant contact on the buccal and palatal/lingual aspect (ΔBICb: −0.26 mm; −0.44 to 0.00, *p* = 0.005 and ΔBICp: −0.46 mm; −0.44 to 0.00, *p* = 0.018) (Table 2 and Figure 3). The analysis was also repeated after excluding the 5 GBR cases (Table S1), but the outcomes remained consistent.

**TABLE 2:** Peri‐implant radiographic bone levels as assessed through CBCT scan.

	Bw‐0	Bw‐1	Bw‐2	Bw‐4	Pw‐0	Pw‐1	Pw‐2	Pw‐4	BICb	BICp
Implant placement	1.05 [0.74 to 1.64]	1.26 [0.92 to 1.69]	1.49 [0.97 to 2.14]	2.05 [1.32 to 2.52]	1.01 [0.66 to 1.51]	1.42 [1.05 to 2.06]	1.84 [1.47 to 2.80]	2.98 [1.93 to 3.35]	0.00 [0.00 to 0.00]	0.00 [0.00 to 0.00]
12 months post‐loading	0.00 [0.00 to 1.48]	1.24 [0.72 to 1.94]	1.36 [0.99 to 2.28]	1.95 [1.36 to 2.59]	0.52 [0.00 to 1.21]	1.24 [0.96 to 1.90]	1.41 [1.22 to 2.65]	2.67 [1.65 to 3.54]	0.41 [0.00 to 0.58]	0.00 [0.00 to 0.46]
Change	0.53 [0.21 to 1.00]	0.20 [−0.02 to 0.30]	−0.01 [−0.13 to 0.27]	0.14 [−0.14 to 0.37]	0.47 [−0.04 to 0.90]	0.12 [−0.05 to 0.51]	0.06 [−0.11 to 0.52]	0.02 [−0.17 to 0.37]	−0.26 [−0.44 to 0.00]	−0.46 [−0.44 to 0.00]

*Note:* Data are expressed as median and interquartile range [Q1–Q3]. The third row (in grey) showed the change in bone levels between implant placement and 12 months post loading.

Abbreviations: BIC, vertical distance between the implant shoulder and the first bone to implant contact; Bw, buccal width; Pw, palatal/lingual width.

A positive correlation was obtained between ΔBICb and T score (*r* = 0.775, *p* < 0.001), previous history of periodontal treatment (*r* = 0.573, *p* = 0.016), baseline FMBS and the fact that GBR was performed (*r* = 0.486, *p* = 0.048 and *r* = 0.587, *p* = 0.013, respectively). This means that vertical bone levels were more stable at 1 year post‐loading in patients who had less severe osteoporosis, received previous periodontal treatment, had initial higher FMBS, and received GBR concomitant to implant placement. ΔBw‐0 negatively correlated with previous history of periodontal treatment (r = −0.712, *p* = 0.001) and complexity of implant case (i.e., presence of dehiscence or fenestration) (r = −0.537, *p* = 0.026), meaning that the buccal regenerated bone was more stable in patients that received previous periodontal treatment and when GBR was performed to treat dehiscence or fenestration osseous defects. In all GBR‐treated cases, the fenestration/dehiscence was covered at 12 months.

### 3.2 Periodontal and Peri‐Implant Clinical Parameters

The mean FMPS and FMBS at baseline were 19.69% ± 10.26% (min 5%, max 39%) and 9.98% ± 9.01% (min 0%, max 31%), respectively, with an average number of 3.6 missing teeth. Only one patient was withdrawn after visit 1 because it was not possible to achieve an adequate level of periodontal health (FMPS > 70% and presence of residual pockets) after multiple rounds of non‐surgical periodontal therapy.

None of the patients completing the study required surgical periodontal therapy, but they all required at least one extra visit of non‐surgical therapy either for supra/subgingival instrumentation or just for oral hygiene instructions and to improve FMPS and FMBS.

The mean implant PPD at 6 and 12 months of follow‐up was 2.98 ± 0.81 mm (mean ranges: min 2.17 mm, max 5.33 mm) and 2.77 ± 0.87 mm (mean ranges: min 1.33 mm, max 4.33 mm), respectively, while recession was 0.05 ± 0.17 mm (mean ranges: min 0 mm, max 0.67 mm) and 0.07 ± 0.25 mm (mean ranges: min 0 mm, max 1 mm). The mean bleeding score around the implant was 19.60% ± 23.74% (mean ranges: min 0% max 83.33%) at 6 months and 21.87% ± 20.83% (mean ranges: min 0%, max 66.67%) at 12 months post‐loading, respectively. The implants that had no bleeding or ≤ 1 spot of bleeding were 10 at 6 months and 8 at 12 months. Conversely, peri‐mucositis was diagnosed in 7 implants at 6 months and 8 implants at 12 months (clinical data were not available for 1 implant at 6 months and for 2 implants at 12 months). The mean plaque score around the implant was 9.80% ± 17.74% (mean ranges: min 0%, max 50%) at 6 months and 16.67% ± 20.21% (mean ranges: min 0%, max 66.67%) at 12 months post‐loading, respectively.

At the 6‐month post‐loading follow‐up, one patient presented with pus discharge from the implant site and a PPD of 7 mm. After removing the crown, a piece of floss was retrieved, which most probably had acted as a foreign body, thus causing the acute inflammation. After performing debridement, irrigation with chlorhexidine and providing instructions to the patients on how to use interdental brushes rather than floss, the crown was screwed back in place and the patient regularly re‐assessed. At the 12‐month post‐loading visit, no inflammation was detected, and PPD was reduced, with only one site presenting PPD of 5 mm, but no BOP.

### 3.3 RFA Analysis

**FIGURE 4:**
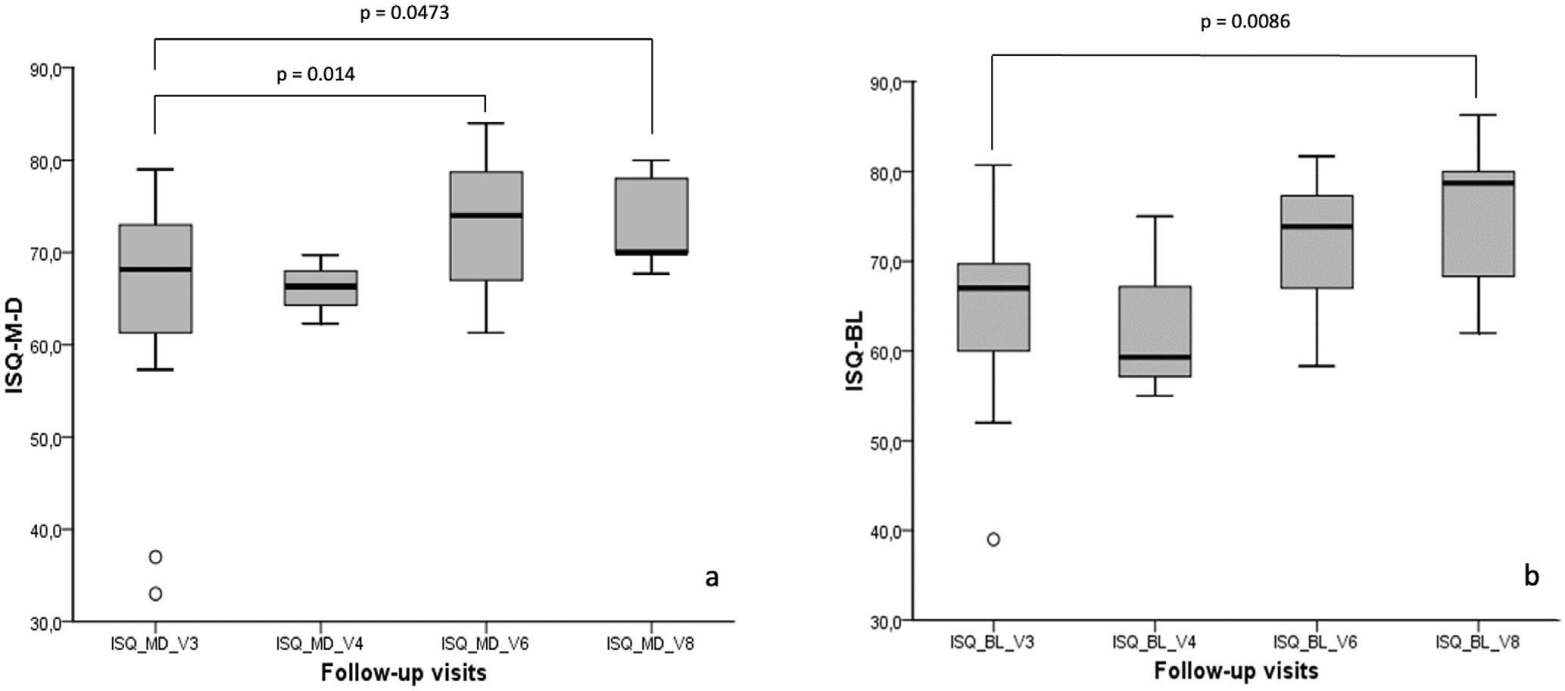
Boxplot representing ISQ changes measured in the mesio‐distal direction (a) and bucco‐lingual (b) direction at the different time points.

After a non‐significant decrease at 1 week after implant placement (visit 4), ISQ progressively increased at the subsequent follow‐ups, both for the mesio‐distal recording and bucco‐lingual recording (*p* = 0.0081 and *p* = 0.0041, respectively) (Figure 4). In particular, the Dunn test showed a significant change from visit 3 to visit 6 for mesio‐distal ISQ (*p* = 0.014) and from visit 3 to visit 8, both for mesio‐distal and bucco‐lingual ISQ (*p* = 0.0473 and *p* = 0.0086, respectively). We did not observe any significant difference in both ISQ mesio‐distal and bucco‐lingual recordings between visit 3 and other time points.

### 3.4 Implant Survival and Success

All implants were in place at 12 months post‐loading (100% survival rate, 95% CI: 81.5%–100%) and they all fulfilled Buser et al. (Buser et al. 1990) and Albrektsson et al. (Albrektsson et al. 1986) criteria for implant success (100% success; 95% CI: 81.5%–100%). When applying Ong et al. (Ong et al. 2008) criteria, the success rate was 81.3% (95% CI: 54.4%–96%), since three implants presented at least one site with PPD ≥ 5 mm and BOP at 12 months post‐loading.

## 4 Discussion

This is the first prospective case series describing the use of a hydrophilic titanium‐zirconium alloy implant for the rehabilitation of osteoporotic patients.

The study showed that, even in the presence of osteoporosis, it is possible to achieve high short‐term survival and success rates when implants with this specific surface are employed. Furthermore, the results suggest that there is no need for adjusting the implant placement and loading protocols suggested by the manufacturer, with stable radiographic peri‐implant bone levels at 12 months post loading. As a matter of fact, despite the presence of osteoporosis, healing time before loading was kept at 8 weeks (12 weeks in case GBR was performed) and osseointegration was successfully obtained in all cases. Moreover, after an expected slight decrease in the week after implant placement, ISQ significantly increased at loading (8 weeks after implant placement) and at the final follow‐up visit (12 months post loading) (Figure 4), which is in line with what reported in previous studies and suggested a successful and timely osseointegration (Han et al. 2010). Nevertheless, it should be highlighted that our findings strictly apply to this controlled cohort of osteoporotic patients and we cannot generalize on the impact that this implant surface may have on reversing the effect of osteoporosis.

Previous systematic reviews found comparable implant survival rates in healthy and osteoporotic patients (de Medeiros et al. 2018; Holahan et al. 2008), thus indicating that implant treatment is a viable option also in the presence of osteoporosis. Nevertheless, Temmerman et al. (Temmerman et al. 2017) reported an increased peri‐implant marginal bone loss in osteoporotic patients compared to healthy patients at 12 months post loading both at implant (−0.11 ± 0.49 mm vs. 0.05 ± 0.52 mm) and patient level (−0.17 ± 0.30 mm vs. 0.04 ± 0.23 mm). They also suggested a different pattern of bone loss, with osteoporotic patients losing most of the bone after loading, while control patients tended to lose peri‐implant bone mainly before loading. Likewise, an older study on patients receiving implant‐supported overdentures showed increased 5‐year radiographic peri‐implant bone loss in patients with a T score < −2 compared to patients with a T score > −2 (0.47 mm vs. 0.01 mm) (von Wowern and Gotfredsen 2001).

In the present study, despite the level of buccal/lingual width at implant platform and the vertical bone levels were significantly different from visit 3 to 12 months of follow‐up, such changes can be considered as clinically non‐relevant and probably part of the physiologic peri‐implant bone remodelling that occurs after loading (Papaspyridakos et al. 2012; Roos‐Jansaker et al. 2006; Tarnow et al. 2000) (Figure 3). Moreover, they are in line with the mean 2‐D radiographic marginal bone loss at 1 year reported in a systematic review on titanium‐zirconium dental implants (0.36 ± 0.06 mm) (Altuna et al. 2016). To the best of our knowledge, only one study previously applied CBCT scans to assess the stability of peri‐implant bone levels in osteoporotic patients, and it showed a non‐significant reduction in alveolar width at 9 months post‐implant placement combined with GBR in 10 subjects (Tadinada et al. 2015). It should be noted that in cases where GBR was performed, the buccal measurements did not necessarily reflect the stability of the pristine bone, but the stability of a mixture of pristine bone and the biomaterials employed for GBR. The use of a collagen‐based membrane and xenograft was a safe and predictable treatment (Calciolari et al. 2023).

It is also important to mention that, while CBCTs are the only radiographic tool that allows the tri‐dimensional assessment of peri‐implant bone levels, they also present with limitations and their image quality varies in relation to the device, the different technical parameters, and the possibility of having artifacts (Pelekos et al. 2018; Schriber et al. 2020). It is also important to mention that it is not standard of practice to perform CBCTs after implant placement and at subsequent follow‐ups to assess bone level changes, but this was done for research purposes. Nevertheless, the use of new generation CBCT scans and new scanning protocols, which reduce the amount of radiation and are only focused on the region of interest, helped reduce the delivered dose.

Remarkably, in the current study, vertical bone resorption correlated with T score, meaning that it appeared that less bone loss occurred in patients with less severe osteoporosis, as well as with the performance of GBR, meaning that when bone regeneration was performed, this allowed a better stability of peri‐implant vertical bone levels. Likewise, buccal bone levels were more stable in complex cases with dehiscences/fenestrations, since these were the situations in which GBR was performed. Future larger studies are needed to confirm these findings and explore the correlation of peri‐implant bone stability with other potentially relevant parameters, such as intake of osteoporosis medications or implant location.

A high survival and success rate was obtained at 12‐month follow‐up. When looking at less stringent criteria (Albrektsson et al. 1986; Buser et al. 2009), a 100% success rate was obtained, while when applying more strict criteria that considered also PPD and BOP (Ong et al. 2008), a lower success rate was observed (81.3%; 95% CI: 54.4%–96%). A previous systematic review on the performance of titanium–zirconium implants indicated a mean survival and success rate of 98.4% and 97.8% at 1 year after implant placement, but it is unclear which parameters were considered to define implant success or if these were consistent among included studies (Altuna et al. 2016).

Although it was not within the remit of this study to assess the quality of jawbone in osteoporotic patients, it should be noted that in 4 out of 18 implants, the torque did not reach 15 Ncm, and in 2 cases, spinning of the implants occurred. Moreover, the high drop‐out rate after visit 1 was mainly due to the presence of extensive alveolar ridge resorption that prevented implant placement without staged GBR or sinus lift. Although it is still controversial whether osteoporosis has a detrimental effect on the jawbones, growing evidence from pre‐clinical and clinical studies seems to confirm a correlation between bone density measured at different systemic skeletal sites and at the jawbones (Anwar et al. 2007; Drozdzowska et al. 2002; Erdogan et al. 2009; Esfahanizadeh et al. 2013; Horner et al. 1996; Jonasson et al. 2001; Makker et al. 2012; Takaishi et al. 2005; Vishwanath et al. 2011), and that osteoporosis is associated with a reduced bone quality and increased cortical porosity in the jaws (Du et al. 2016; Dvorak et al. 2011; Hirai et al. 1993; Kosugi et al. 2013; Kuroda et al. 2003; Singhal et al. 2012).

Besides the reduced bone quality, it is also likely that the microarchitectural changes in bone structure and impaired osseous healing associated with osteoporosis might have a negative impact on the osseointegration process (Donos et al. 2023). In a pre‐clinical model of GBR, we previously reported a tendency for an enhanced inflammatory and stress response and a delayed organization and maturation of the granulation tissue in osteoporotic conditions (Calciolari, Mardas, Dereka, Anagnostopoulos, et al. 2017; Calciolari, Mardas, Dereka, Kostomitsopoulos, et al. 2017). In light of this, the use of a hydrophilic micro‐rough implant surface that has been shown to down‐regulate early inflammatory pathways and enhance osteogenesis by upregulating pathways involved in osteoblast differentiation and angiogenesis (Calciolari, Hamlet, et al. 2018; Calciolari, Mardas, et al. 2018; Donos, Hamlet, et al. 2011) might be of particular value to counteract the negative impact of osteoporosis on osseous formation and osseointegration. Previous pre‐clinical studies demonstrated that implant topography and hydrophilicity can compensate for the negative impact of osteoporosis on early osseointegration (Du et al. 2016; Mardas et al. 2011; Siqueira et al. 2021) and the current study follows this line of research and should be seen as a proof of principle study documenting the possibility of successfully treating a category of potentially challenging patients without the need for adjusting standard treatment protocols.

While there is insufficient evidence to establish a potential connection between anti‐resorptive medications and implant failure (Jung et al. 2023), the risk of developing implant surgery‐triggered medication‐related osteonecrosis of the jaw (MRONJ) cannot be ruled out (Sher et al. 2021). Despite there is no established guideline on the best timing to place implant in patients taking bisphosphonates for osteoporosis or on the need to have a drug holiday, in the current project we decided to exclude patients in treatment for more than 4 consecutive years, based on the study by Lo et al. (Lo, et al. 2010) that stratified the risk of MRONJ by treatment duration and indicated an increased prevalence in patients with ≥ 4 years of medication exposure compared with those with < 4 years of exposure (0.21% vs. 0.04%, respectively).

While no cases of MRONJ developed in the current study, it is important that osteoporotic patients taking bisphosphonates who are undergoing invasive surgical procedures, including implant placement associated or not with bone regeneration, are adequately informed of the risk, albeit small, of developing MRONJ and that clinicians take all necessary precautions to make the surgeries as less invasive as possible and promote healing for primary closure (Al‐Nawas et al. 2023; American Dental Association Council on Scientific 2006).

This study presented some shortcomings. Due to the proof‐of‐principle nature of the case series, we lacked a control group of healthy patients. However, previous studies already documented the use of this titanium‐zirconium alloy implant with a hydrophilic surface for fixed and removable prosthetic rehabilitations in non‐osteoporotic patients (Chiapasco et al. 2012; Ioannidis et al. 2015; Quirynen, et al. 2015), and our radiographic outcomes at 12 months post‐loading are in line with those studies. While using CBCTs to assess peri‐implant bone levels is an established methodology in the literature, and we used the same machine with the same settings in all patients, it should also be highlighted that the beam‐hardening artifact associated to the presence of implants and poor soft tissue contrast make it challenging to assess small bone level changes. Moreover, the examiner assessing peri‐implant bone parameters could not be blinded since the CBCTs performed at 12 months were always done with the implant crown in place. Therefore, they were easily distinguishable from the CBCTs performed after implant placement. Nevertheless, scans were assessed in random order (not based on the patient) to reduce the risk of bias.

In this case series, both maxillary and mandibular implants were placed, and we included implants to replace anterior as well as posterior teeth (up to first molars), therefore, we cannot make any assumption on a possible differential impact of the underlying osteoporosis based on implant location. However, it is well‐known that in osteoporotic subjects, bone loss is not uniform, and that trabecular bone is earlier and more deeply affected than cortical bone (Clarke and Khosla 2010; Khosla 2013). Hence, it is likely that the reduced bone density caused by osteoporosis might be more evident in the maxilla, particularly in the anterior area (Esfahanizadeh et al. 2013; Gulsahi et al. 2010). Future studies are needed to explore this hypothesis.

The population was heterogeneous in terms of medication consumption, with 55.56% of the patients in current treatment with antiresorptive medications (for < 4 years), 11.11% previously treated, and 33.33% that never took medications. In future studies, it would be interesting to investigate the effect of the accumulated dose of antiresorptive medications on the stability of peri‐implant bone levels.

Finally, we combined cases that underwent GBR concomitant to implant placement and cases where no GBR was performed. While this is a limitation and makes the population more heterogeneous, excluding cases in need of GBR would have made it challenging to complete recruitment, as a potential increased bone resorption may be expected in osteoporotic patients. Remarkably, we found a significant positive correlation between ΔBICb and the fact that GBR was performed and a negative correlation between ΔBw‐0 and the complexity of the implant case (i.e., presence of dehiscence or fenestration, which would require GBR). However, it is not possible to draw robust conclusions on the benefit of performing GBR to preserve and maintain peri‐implant bone levels, considering the limited number of GBR cases treated and the lack of power to answer this question.

In conclusion, despite this case series being exploratory in nature and no sample size calculation being performed, it could be suggested that the use of a titanium‐zirconium implant with a micro‐rough hydrophilic surface in patients affected by osteoporosis could lead to predictable short‐term (12 months post loading) implant outcomes, even in the case of current antiresorptive therapy (for < 4 years).

Future studies are warranted to assess the stability of peri‐implant bone levels and the success/survival of implant rehabilitations in osteoporotic patients in the longer term. Based on the results of this case series study, there is now the possibility to properly power a future prospective controlled study that can clarify whether the application of such an implant surface that is aimed at enhancing osseointegration by modulating specific signaling pathways can promote comparable implant outcomes in osteoporotic and healthy patients, without the need for adjusting the protocol healing time and without incurring in an increased risk of adverse events or failures.

## Author Contributions

**Calciolari Elena:** writing – original draft, writing – review and editing, investigation, methodology, formal analysis. **Mardas Nikos:** investigation, methodology, writing – review and editing. **Palaska Iro:** investigation, writing – review and editing, methodology. **Tagliaferri Sara:** formal analysis, data curation, writing – review and editing. **Donos Nikolaos:** conceptualization, investigation, methodology, writing – original draft, writing – review and editing, project administration, funding acquisition.

## Ethics Statement

The study was approved by the London Bridge Research Ethics Committee on 29th April 2016 (16/LO/0477).

## Consent

All patients signed an informed consent form before undertaking any study‐related visit.

## Conflicts of Interest

Professor Nikolaos Donos received an Investigator‐led grant from Institut Straumann AG (Basel, Switzerland) that supported this study.

## Supporting information


Appendix S1.

## Data Availability

The data that support the findings of this study are available from the corresponding author upon reasonable request.
