# Supplementary material for: Radiographic Peri‐Implant Bone Changes in Osteoporotic Women Treated With a Ti‐Zr, Bone Level Tapered Implant With a Hydrophilic Surface: A 12‐Month Prospective Case‐Series
Source: Clin Oral Implants Res. 2025 Jul 8;36(10):1234–47. doi: 10.1111/clr.14469 (PMC12491925; doi:10.1111/clr.14469)
Supplement: Supplementary file 1 — Appendix S1. [file CLR-36-1234-s001.docx]

Supplementary material

Figure S1: a, representation of the reference points employed to assess radiographic peri-implant bone changes in a case where GBR was not performed (a) and in a case where GBR was performed (b). In the latter, the buccal regenerated bone is clearly demarcated with a pink line to facilitate visualization.

Table S1: Peri-implant radiographic bone levels as assessed through CBCT scan after excluding GBR cases. Bw, buccal width; Pw, palatal/lingual width; BIC, vertical distance between the implant shoulder and the first bone to implant contact. Data are expressed as median and interquartile range [Q1-Q3].

|  | Bw-0 | Bw-1 | Bw-2 | Bw-4 | Pw-0 | Pw-1 | Pw-2 | Pw-4 | BICb | BICp |
| --- | --- | --- | --- | --- | --- | --- | --- | --- | --- | --- |
| Implant placement | 1.00 [0.58-1.27] | 1.10 [0.80-1.36] | 1.14 [0.93-1.82] | 1.87 [1.22-2.46] | 1.01 [0.66-1.51] | 1.37 [1.00-1.79] | 1.70 [1.38-2.72] | 2.48 [1.81-3.08] | 0.00 [0.00-0.00] | 0.00 [0.00-0.00] |
| 12 months post loading | 0.00 [0.00-0.37] | 1.04 [0.49-1.44] | 1.21 [0.83-1.85] | 1.80 [1.16-2.42] | 0.55 [0.00-1.21] | 1.24 [1.02-1.90] | 1.41 [1.22-2.65] | 2.55 [1.65-3.06] | 0.44 [0.13-0.73] | 0.00 [0.00-0.58] |
| Change | 0.62 [0.28-1.03]  **p=0.002** | 0.20 [-0.02-0.29]  **p=0.101** | -0.02 [-0.21-0.27]  **p=0.753** | 0.14 [-0.12-0.33]  **p=0.463** | 0.49 [-0.06-0.90]  **p=0.033** | 0.10 [-0.14-0.40] **p=0.249** | 0.05 [-0.13-0.52]  **p=0.311** | 0.04 [-0.12-0.37]  **p=0.249** | -0.43 [-0.58-0.06]  **p=0.005** | 0.00 [-0.58-0.00]  **p=0.028** |
